# Simultaneous Enhancement of Strength and Toughness of PLA Induced by Miscibility Variation with PVA

**DOI:** 10.3390/polym10101178

**Published:** 2018-10-22

**Authors:** Yanping Liu, Hanghang Wei, Zhen Wang, Qian Li, Nan Tian

**Affiliations:** 1 School of Mechanics and Engineering Science, Zhengzhou University, Zhengzhou 450001, China; ypliu@zzu.edu.cn (Y.L.); hanghang_wei@163.com (H.W.); 2 National Centre for International Joint Research of Micro-nano Molding Technology, Zhengzhou University, Zhengzhou 450001, China; 3 National Engineering Research Center for Advanced Polymer Processing Technology, Zhengzhou University, Zhengzhou 450002, China; wangz@zzu.edu.cn; 4 MOE Key Laboratory of Space Applied Physics and Chemistry, Shanxi Key Laboratory of Macromolecular Science and Technology, School of Science, Northwestern Polytechnical University, Xi’an 710072, China

**Keywords:** poly (vinyl alcohol), poly (lactic acid), miscibility, mechanical property

## Abstract

The mechanical properties of poly (lactic acid) (PLA) nanofibers with 0%, 5%, 10%, and 20% (*w*/*w*) poly (vinyl alcohol) (PVA) were investigated at the macro- and microscale. The macro-mechanical properties for the fiber membrane revealed that both the modulus and fracture strain could be improved by 100% and 70%, respectively, with a PVA content of 5%. The variation in modulus and fracture strain versus the diameter of a single electrospun fiber presented two opposite trends, while simultaneous enhancement was observed when the content of PVA was 5% and 10%. With a diameter of 1 μm, the strength and toughness of the L95V5 and L90V10 fibers were enhanced to over 3 and 2 times that of pure PLA, respectively. The structural evolution of electrospun nanofiber was analyzed by differential scanning calorimetry (DSC) and Fourier transform infrared spectroscopy (FTIR). Although PLA and PVA were still miscible in the concentration range used, the latter could crystallize independently after electrospinning. According to the crystallization behavior of the nanofibers, a double network formed by PLA and PVA—one microcrystal/ordered structure and one amorphous structure—is proposed to contribute to the simultaneous enhancement of strength and toughness, which provides a promising method for preparing biodegradable material with high performance.

## 1. Introduction

Over the past several decades, poly (lactic acid) (PLA) has attracted increasing attention because of its biological properties and renewability [1,2]. PLA products with good mechanical strength are widely used, especially as fixation devices in the biomedical field, such as sutures, pins, scaffolds, and drug delivery devices [3]. However, the applications of PLA-based products are often restricted due to the lack of suitable mechanical properties, such as flexibility and plasticity. Many studies have been conducted to improve the mechanical properties, and the most common way is by adding plasticizers or other polymeric materials to PLA, such as kenaf fibers, wood pulp, cassava, poly(ε-caprolactone), polyethylene glycol, and so on [4,5,6,7,8,9,10]. The strength or toughness can be improved by adding polymer materials, but the simultaneous enhancement is still a challenge. Moreover, two additional key issues that need to be considered further when choosing reinforcing material for use in biomedical applications are nontoxicity and complete biodegradability. In brief, the selection of an additive for a specific PLA composition requires the consideration of many criteria: compatibility, low volatility, strength, toughness, degradability, lack of toxicity, and so on [11].

As a water-soluble polymer, poly (vinyl alcohol) (PVA) has been used in many fields, such as microcapsules of pharmaceuticals, film-forming materials, controlled-release drugs, and crosslinking agents of tissue engineering materials [12,13,14,15]. In addition to its chemical and thermal stability, PVA is also a hydrophilic, nontoxic, and noncarcinogenic polymer with excellent mechanical properties [16,17]. Therefore, PVA is an excellent choice for improving the properties of PLA. More importantly, stronger hydrogen bonding is formed between the hydroxyl groups of PVA and the oxygen atom of the ester groups in the PLA chain, which contributes to the miscibility or partial miscibility [18,19,20,21,22]. Compatibility between PVA and PLA can be improved by stannous octoate, providing a better tensile property in terms of elongation at break [23]. A conventional melt-spinning method was used to prepare a PVA/PLA nanofibrillary structure by Tran et al. [24,25,26], although phase separation induced by spinning was found. PLA 2D and 3D textile structures were produced when the matrix component PVA was removed in water at the expense of mechanical properties.

For the application in a biomedical field, nanofiber scaffold has been considered as a potential scaffold material, because electrospun nanofibers can imitate biomimetic nanostructures which may act as proxies of the native tissue [27,28,29]. Also, the topographical and biochemical cues that promote tissue healing can be provided. Raquel et al. obtained core–shell PVA/PLA ultrafine fibers by coaxial electrospinning, and the hydrophilicity of pure fibers was improved obviously, indicating a good potential for use as biomaterials for the controlled release of bioactive molecules [30]. With reduced size and dimensionality, polymer nanofibers exhibit exceptional mechanical properties compared with those of their macroscopic counterparts, since the former one is better treated as a one-dimensional system. A supramolecular structure with oriented macromolecules is estimated to be formed in the amorphous zone of nanofibers, which contributes greatly to the deformation process [31]. Recently, a cylinder-like structure was found by TEM and AFM in amorphous polycarbonate nanofibers, confirming the supramolecular structure [32]. Therefore, it is worthwhile to focus on the complex structural evolution of composite fibers.

In this study, a composite nanofiber of PLA/PVA with different PVA content (0%, 5%, 10%, 20% (*w*/*w*)) was fabricated by electrospinning. The morphology, thermal properties, and macro-mechanical properties were investigated comprehensively. Moreover, single fibers were prepared by a home-made collector, and their properties were obtained from tests on a nanomechanical testing system, which provides information on a smaller scale.

## 2. Materials and Methods

### 2.1. Materials

The PLA (4032D, Nature Works) used in this study has a weight-averaged molecular weight (*Mw*) of 2.1 × 10^5^ g mol^−1^, and the polydispersity is 1.7. The *Mw* of PVA (1799, Tianjin Kermal co. Tianjin, China) is 7 × 10^4^ g mol^−1^. The solvent of hexafluoroisopropanol (HFIP) was used as received.

### 2.2. Electrospun PLA/PVA Nanofibers

PLA and PVA at different weight ratios of 100/0, 95/5, 90/10, 80/20 (*w*/*w*) were added to HFIP under stirring to prepare a 6% (*w*/*v*) solution at room temperature. The solution was stirred for 12 h so that it was well dissolved. Electrospinning was carried out on a standard electrospinning apparatus, and the collector plate was fixed below the needle tip with a working distance of 20 cm. A needle with an inner diameter of 0.9 mm and voltage of approximately 16 kV were used. Furthermore, to ensure the uniformity of the film thickness, a roller collector was equipped with a speed of 300 r/min. In this way, electrospun samples with a uniform thickness were obtained and the error in mechanical properties caused by sample inhomogeneity could be thus maximally minimized. All samples were dried for 24 h at 37 °C under vacuum after electrospinning.

### 2.3. Characterization

The morphology of the fiber membranes was analyzed by scanning electron microscopy (SEM, Keysight 8500FE, Santa Rosa, CA, USA). Differential scanning calorimetry (DSC, TA Q2000, New Castle, DE, USA) was used to measure the thermal properties. The sample was first heated to 200 °C from 0 °C at 10 °C/min. After 5 min at 200 °C, it was cooled at 10 °C/min to 0 °C, followed by a second heating and cooling round under the protection of nitrogen. Fourier transform infrared spectroscopy (FTIR) was performed using the Nicolet 6700 (Watertown, MA, USA) analyzer.

### 2.4. Mechanical Testing

An electronic universal testing machine (SUNS, UTM 2203, Shenzhen, China) was used for the tensile tests. Specimens were prepared by cutting the fiber membrane into 40 × 10 mm strips along the rotation direction. The samples were stretched to fracture with a constant stretching rate of 2 mm/min. Nanomechanical testing of single fibers was performed using a testing system (Keysight T150UTM, Santa Rosa, CA, USA) at a stretching rate of 2.7 × 10^−3^ mm/s, with a load resolution of 50 nN. Twenty samples of single composite fibers were prepared for every weight ratio to satisfy the wide distribution of the diameters. All the samples were 5 mm in initial length and the diameters were separately measured from the initial fiber.

## 3. Results and Discussions

**Figure 1:**
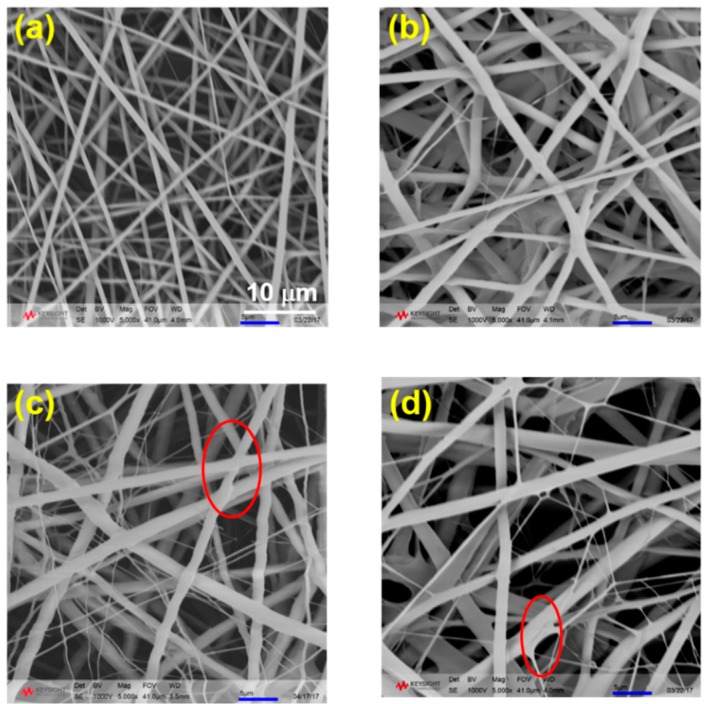
The morphology of poly (vinyl alcohol)/poly (lactic acid) (PVA/PLA) fibers with different weight ratios: (**a**) PLA; (**b**) L95V5; (**c**) L90V10; (**d**) L80V20. The magnification is 5000×.

Considering the fiber quality, four weight ratios between PLA and PVA were chosen as 100:0, 95:5, 90:10, and 80:20, which are denoted as PLA, L95V5, L90V10, and L80V20, respectively. The electrospinning parameter was regulated until the fiber was apparently smooth. The fiber morphology and dimension are influenced by many factors, such as the distance between tip and collector, solvent viscosity, dielectric constant, and so on. The morphology of the fibers with four different ratios was first checked by SEM, as shown in Figure 1. It can be seen that the quality (which mainly refers to the uniformity of the diameter) of the fiber membrane declines as the content of PVA increases. The diameter of pure PLA fiber in Figure 1a is uniform and lower than 1 μm. Electrospun under same conditions, the fibers gradually thicken as more PVA is added. As displayed in Figure 1d, where the weight ratio of PVA is as high as 20%, the fiber dimension is as big as 2 μm. Moreover, many ultrafine fibers appear, and part of the fiber crossing is spliced like welding when the content of PVA is 10% and 20%, as marked in Figure 1c,d.

**Figure 2:**
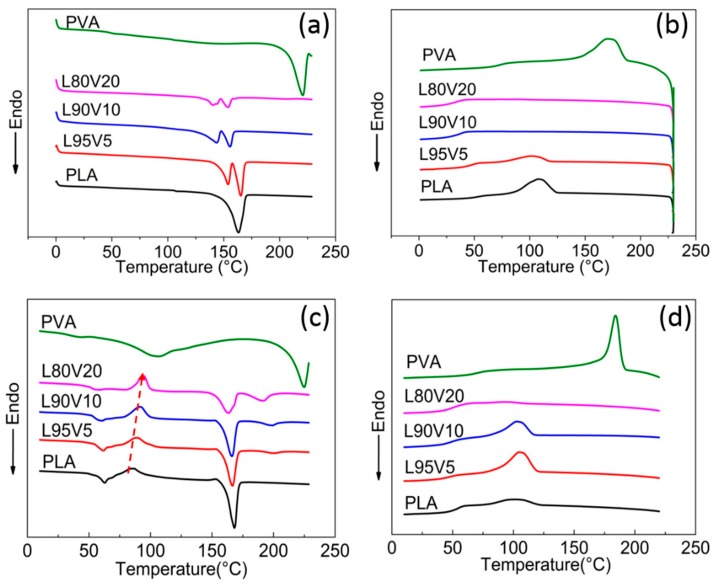
The heating (**a**) and cooling (**b**) differential scanning calorimetry (DSC) curves of the casting film. The first heating (**c**) and cooling (**d**) DSC curves of the PLA fiber membrane with varying content of PVA.

To analyze the structural evolution during electrospinning, the thermal behavior of PVA/PLA blends was investigated first by DSC, as shown in Figure 2a,b. The blends of PLA and PVA are the precipitate from the solution after evaporating the solvent in a culture dish. Before testing, the casting film was also dried at 37 °C for 24 h in a vacuum oven. On the heating curve for pure PLA, only one endothermic peak appears at 168 °C, which is the melting point (*T*_m_) of PLA crystal. The excellent mobility of the molecular chain in the solution contributes to the full crystallization. With the addition of PVA, the mobility of the PLA chains declines due to the interaction that occurs, leading to a decrease in melting point and shoulder peak on the DSC curves of the blends. The melting peak of PVA, which is about 220 °C according to the DSC curve, is absent from the heating curves of the blend, reflecting the good miscibility between the two molecular chains. Moreover, the increased interaction between PVA and PLA hinders the crystallization of PLA, which can also be found from the cooling curves displayed in Figure 2b. With a cooling rate of 10 °C/min, the exothermic peak appears at 115 °C on the PLA cooling curve. When only 5% (*w*/*w*) PVA is blended, the crystallization of PLA is still identifiable at a similar temperature, while no exothermic peak is visible on the cooling curves of L90V10 and L80V20. Meanwhile, the crystallization of PVA cannot be observed for any of the blends. That is, the PVA chains should be well dispersed in the PLA matrix, without local aggregation.

The DSC heating and cooling curves of pure PLA fibers and PVA/PLA composite fibers are displayed in Figure 2c,d. The first endothermic located at 56 °C and the exothermic peak appearing near 90 °C in Figure 2c are attributed to the glass transition and cold crystallization of PLA, respectively. Note that both peaks are absent from the DSC of the casting film, indicating incomplete crystallization during high-speed electrospinning. The structure is also verified by the broad peak on the WAXD curves, as shown in Figure S1 (Supplementary Materials), which might be diffraction from imperfect crystals or mesophase with conformational ordering. For the reader’s convenience, this is referred to as the microcrystal in the subsequent text. A small endothermic peak, whose intensity increases with PVA content, appears at a temperature higher than the *T*_m_ of PLA on the DSC curves of the composite fiber, which should be the melting of the PVA crystal. Since local enrichment of PVA chains is a prerequisite for crystallization, it can be speculated that the dispersity of PVA in the PLA matrix declines during electrospinning. The dispersity is also reflected by the crystallization behavior, which is indicated by the cooling curves of the fiber membrane as displayed in Figure 2d. With the same cooling rate of 10 °C/min, more obvious exothermic peaks can be identified at 105 °C on the curves of both L95V5 and L90V10. Considering the negligible crystallization of the casting film during the cooling process, the crystallization enthalpy of PLA is attributed to the decreased dispersity.

As indicated by the red arrow in Figure 2c, the peak of cold crystallization (*T*_cc_) shifts to a higher temperature with increasing PVA content. For a more detailed analysis, the thermal properties obtained from the DSC analysis are summarized in Table 1. On the heating curve of L80V20, the *T*_cc_ is 94.7 °C, which is 9.2 °C higher than 85.9 °C of pure PLA, as indicated by the red arrow. Cold crystallization is caused by the movement of adjacent chain segments onto the growing surface and folding, while the melting temperature depends on the chain diffusion and segment folding [33]. Due to the hydrogen bonding between PVA and PLA, the barrier of chain diffusion and transportation of PLA chain segments is increased by the addition of PVA. The hydrogen bonds prevent the folding of the PLA chain to the lamellar surface, which is also in line with the changes in *T*_m_ and *T*_cc_. The small crystal formed during cold crystallization also melts at the melting temperature that follows, which contributes to the melting enthalpy, together with original crystal formed from electrospinning. The calculation of PLA crystallinity (*X_c_*) is according to the formula:

*X_c_* = [(*δH^PLA,m^* − *δH^PLA,cc^*)/(*δH^PLA,m,^°* × *W^PLA^*) × 100% (1)

where *δH^PLA,m^* is PLA’s enthalpy of fusion, *δH^PLA,cc^* is the enthalpy corresponding to cold crystallization, *δH^PLA,m,^°* is the enthalpy of fusion when the polyester is in a 100% crystalline state (*δH^PLA,m,^°* = 93.6 J/g) [34], and *W^PLA^* is the mass fraction of PLA in the mixture. The *X_c_* of PLA is indicated in the right-most column in Table 1; it decreases from 33.8% for pure PLA to 17.3% for L80V20. The downtrend of PLA crystallinity may indicate the suppressing effect of PVA on the crystallization of PLA.

**Figure 3:**
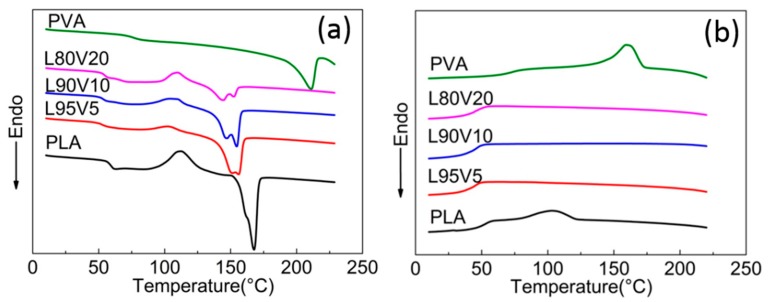
The second heating (**a**) and cooling (**b**) DSC curves of the PLA fiber membrane.

Molecular chains gain greater mobility at elevated temperatures, which will influence the dispersity. Therefore, the second round of the heating and cooling process was carried out for the fiber membrane, as shown in Figure 3a,b. The second heating DSC curves are obviously different from that of the first round shown in Figure 2c, while the melting peak of PLA located near 150 °C is more similar to that of the casting film in terms of the shoulder peak and shift trend. Note that the glass transition temperature (*T*_g_) of the PLA composite is lower than that of PLA (65 °C), which might be due to the interaction caused by hydrogen bonding between PLA and PVA, which enhances the flexibility and mobility of the polymer chains. Because of the faster cooling rate during the first cooling process, cold crystallization and glass transition still present at around 110 °C and 50 °C, respectively. The absence of a higher melting peak on all curves confirms that the dispersity of PVA is improved by melting and recrystallization during the first temperature round. Therefore, the second heating almost eliminates the local aggregation induced by the tensile field of electrospinning. Moreover, there are no exothermic peaks on the second cooling curves of any of the components, as shown in Figure 3b, while the crystallization of the pure PLA fiber is not affected. It can be confirmed that well-dispersed PVA prevents the crystallization of the PLA chains.

**Figure 4:**
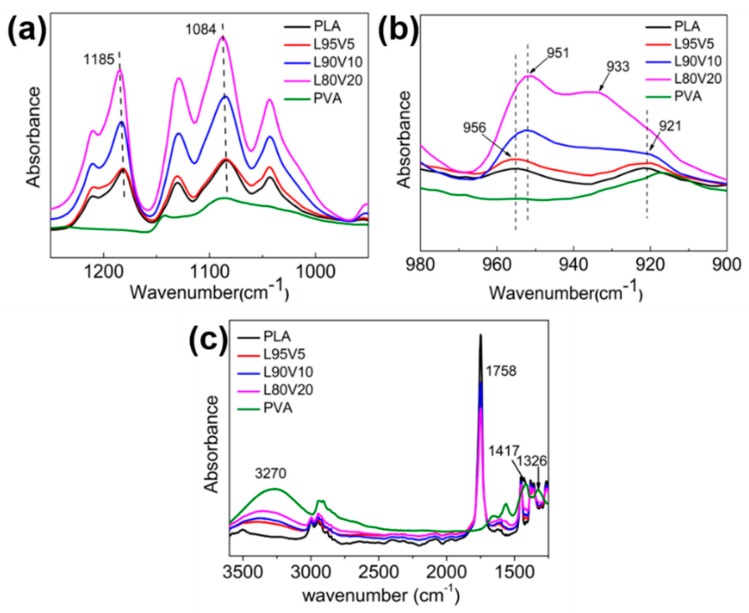
Fourier transform infrared (FTIR) spectra of the pure PLA and PLA/PVA composites in the spectral range of (**a**) 950–1250 cm^−1^; (**b**) 900–980 cm^−1^; and (**c**) 1750–3600 cm^−1^.

FTIR measurement was been performed to analyze the structural changes that might occur during electrospinning. Considering the interaction between PVA and PLA, the characteristic bands related to the hydroxyl groups of PVA and the carbonyl group of PLA which generate intermolecular hydrogen bonding interactions should be checked first in the spectrum. The stretching of the carbonyl group from the PLA crystal sequence corresponds to the band at 1185 cm^−1^, which shifts to a lower frequency with the addition of PVA, as shown in Figure 4a. Correspondingly, the characteristic band of the hydroxyl group of PVA would also transform. The band at 3270 cm^−1^ is assigned to the stretching of the hydroxyl group of PVA, which shifts towards a higher frequency with the decrease in PVA content. The adsorption from hydroxyl bending occurs at 1417 and 1326 cm^−1^, both of which disappear in the composite fiber membrane. Another related band at 1087 cm^−1^ is the stretching of CO and bending of the hydroxyl group from the amorphous sequence of PVA, which also shifts to a higher frequency after blending. It is well known that the stronger hydrogen bond induces a shift in the characteristic bands of the hydroxyl groups and the carbonyl group. The mode stated above indicates the miscibility between PVA and PLA, although the dispersity of the PVA chains declines during electrospinning. Two more famous characteristic bands on the PLA spectrum are 956 and 921 cm^−1^, with the former representing the amorphous sequence. In the region of 900–980 cm^−1^, the band intensity enhances with the increasing content of PVA. The band at 921 cm^−1^ is well assigned to the coupling of the C–C backbone stretching with the CH_3_ rocking mode and is sensitive to the 10_3_ helix chain conformation of the PLA α crystal. The band at 921 cm^−1^ is very weak on the spectrum of L90V10, which is indiscernible when the concentration is 20% (*w*/*w*). Obviously, the crystallization of PLA is suppressed when more PVA is blended, which is also verified by the crystallinity calculated from DSC.

**Figure 5:**
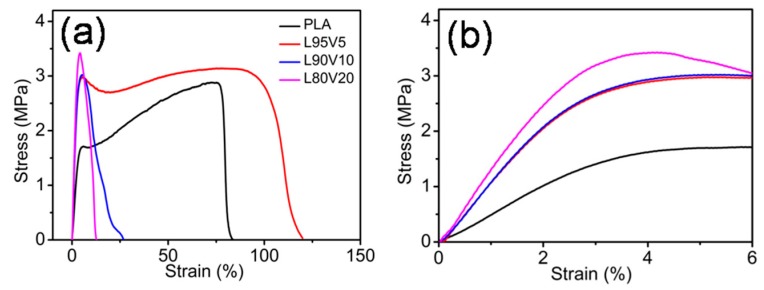
(**a**) Stress–strain curves of electrospun fiber PLA membranes; (**b**) magnified low-strain regions of the stress–strain curves.

The tensile properties of the pure PLA and the PLA/PVA composite fiber membranes was investigated to determine the quasi-static mechanical properties, representing the macro-mechanical properties. The typical stress–strain curves of the fiber membranes are shown in Figure 5a, while Figure 5b is the magnified low-strain regions of the stress–strain curves. It can be observed that PLA and L95V5 exhibit similar stress–strain behavior, which has a linearly elastic region at low strain, followed by the plastic deformation before breaking. The yield stress and fracture strain of pure PLA are 1.6 MPa and 0.8, respectively. When only 5% (*w*/*w*) PVA is blended, both of the two parameters increase, especially the yield stress, which rises to almost 3 MPa. For comparison, Young’s modulus was also calculated from the linear region on the stress–strain curves, as displayed in Table 2. The modulus for the pure PLA membrane is about 50 MPa, which was increased 2-fold by adding 5% or 10% PVA. Besides strength, the toughness also improves, as the fracture strain increases from 0.8 to more than 1. However, as more content of PVA is added, which is displayed as blue and pink lines in Figure 5a, the fracture strain declines sharply to lower than 0.2. Meanwhile, yield strength remains at around 3 MPa, which can be distinguished from the local enlargement in Figure 2b. Therefore, the composite fiber membrane of PLA/PVA is much better than pure PLA in terms of strength, while excessive PVA content (more than 10% (*w*/*w*)) leads to a drop in toughness.

**Figure 6:**
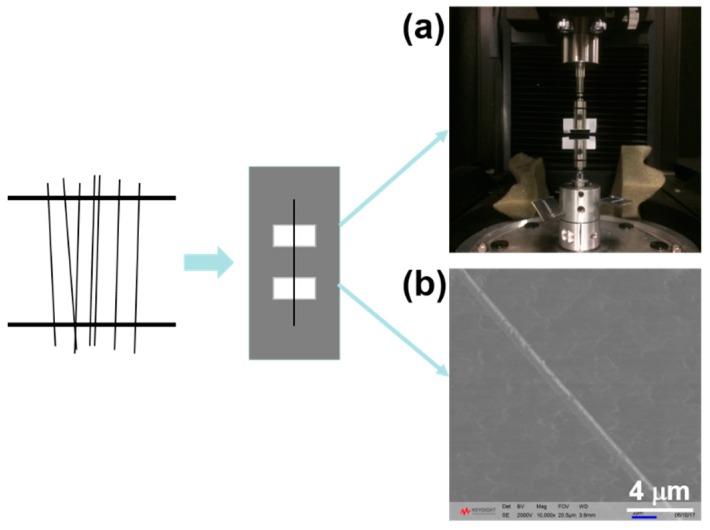
A schematic diagram for the preparation of single fiber samples. (**a**) Setup for the uniaxial tensile testing of a single electrospun fiber; (**b**) SEM morphology of a single electrospun fiber.

It should be noted that a single fiber is essentially different from the common bulk system, which is a three-dimensional system, while the former is better treated as a one-dimensional system [31]. For nanofibers, especially for tissue engineering, not only the macro-mechanical properties should meet the requirement: the micro-mechanical properties also have a great effect on the growth of a cell [35,36,37]. To assess the micro-mechanical response, a home-made collector with a square frame was adopted to collect the nanofibers, as shown in the schematic diagram in Figure 6. A single fiber was carefully removed under bright light and placed on a paper frame with two sections. Because fiber size fluctuates occasionally, even under the same conditions, more than 20 samples with different diameters were prepared for statistical analysis. Half of the fiber with a 5 mm gauge length was used as the tensile test specimen (Figure 6a), and the other half was used for measuring the diameter by SEM (Figure 6b).

**Figure 7:**
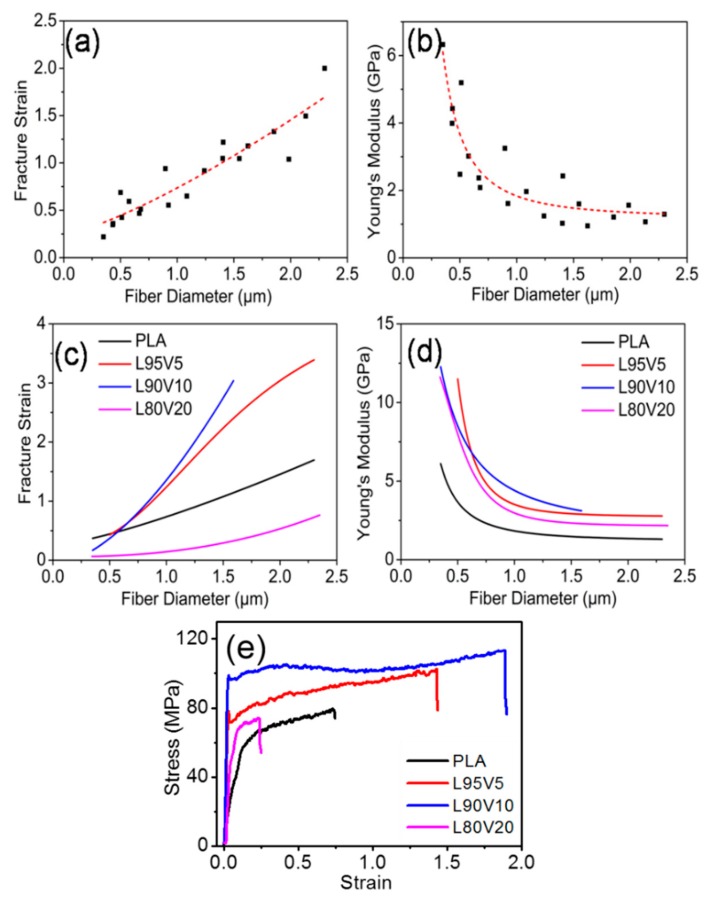
(**a**) Fracture strain and (**b**) Young’s modulus of pure PLA nanofibers with different diameters; (**c**) fracture strain and (**d**) Young’s modulus versus diameter for composite fibers with different PVA content; (**e**) engineering stress–strain curves for single fibers with a diameter of 1 μm.

The micro-mechanical properties of a single fiber were obtained by the nanomechanical testing system. To minimize error from a single specimen, twenty samples were tested to analyze the influence of diameter on the mechanical properties of PLA nanofibers. Figure 7a,b exhibit the change in fracture strain and Young’s modulus of the PLA fiber, with the diameters ranging from 0.3 μm to 2.5 μm. To present this more clearly, the evolution trend versus diameter was fitted by the related function, and the result is displayed as the red dotted line. With fiber thickening, the fracture strain increases almost linearly, from a strain of 0.2 to 2. However, Young’s modulus declines exponentially (Supporting Information), as shown in Figure 7b. When the fiber diameter is in the region from 300 nm to 1 μm, Young’s modulus decreases drastically to 2 GPa from 6.3 GPa. When the diameter is larger than 1 μm, the downtrend gradually slows down.

A similar fitting was also conducted for the PLA/PVA fibers with different ratios, which can be found in Figures S2 and S3. For the convenience of comparison, the fitting curves of the fracture strain and the modulus with different component ratios are plotted in Figure 7c,d, respectively. Obviously, the fiber toughness is improved greatly when 5% or 10% (*w*/*w*) PVA is added. The engineering stress–strain curves of single fibers with a diameter of 1 μm are plotted in Figure 7e; the fracture strains of PLA, L95V5, and L90V10 are 0.7, 1.5, and 1.85, respectively. When the content of PVA increases to 20% (*w*/*w*), the fracture strain is about 0.25. That is, the toughness is improved when the mixture content of PVA is less than 10%, while excess PVA causes a sharp drop in the toughness. From the fitting curves displayed in Figure 7d, it can be seen that all of the modulus versus diameter curves present a nonlinear decline. It should be noted that the modulus of the composite fiber is higher than that of pure PLA, which is independent of the components and fiber diameter. When the diameter is 400 nm, the modulus increases to more than 9 GPa for the blended fiber, while the pure fiber is only 4 GPa. The gap shrinks to less than 2 GPa when fiber diameter increases to 1 μm. The modulus of the electrospun membrane is much lower than that of the single fiber, while the unit of the latter is several GPa. An immediate reason is the difference in transversal size. The fiber membrane is formed by the random stacking of single fibers. The masses of the voids among fibers weakens the mean stress, which is the external force divided by the cross-sectional area. Another key factor is the supramolecular structure, which has been reported to play a dominant role in the deformation process [31]. It is assumed that the segment mobility in the correlated regions of the amorphous parts under confinement with a small diameter is one cause for the increase in Young’s modulus. The polymer ultrafiber possesses an abrupt increase in Young’s modulus when the diameter becomes small enough [38,39].

**Figure 8:**
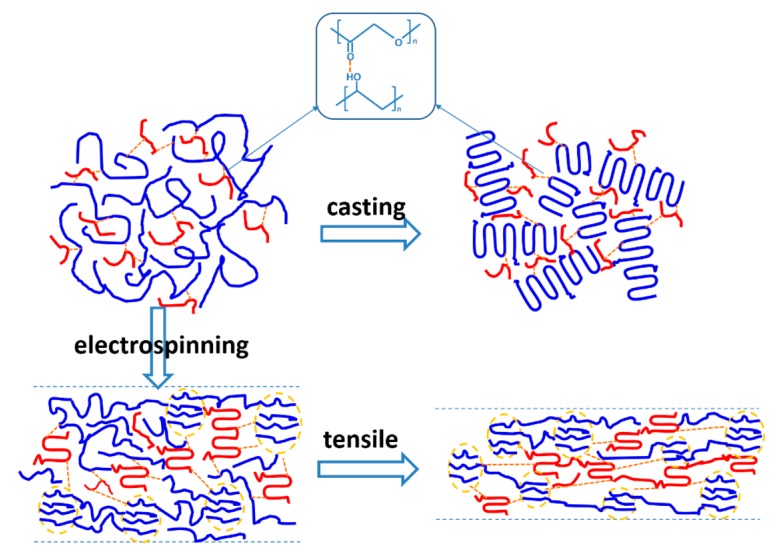
Schematic illustration of the miscibility evolution between PLA and PVA.

Although the micro-mechanical properties are not exactly the same as the macroscopic mechanical properties, both the strength and toughness of PLA could be simultaneously improved by blending with PVA. As a tough material, PVA is considered to enhance the resilience of PLA easily. It should be noted that the tensile properties of the casting film cannot be obtained due to the brittleness of the blended film, which is connected with its internal structure. A schematic illustration of the structure’s evolution is shown in Figure 8, where hydrogen bonding between carbonyl and hydroxyl contributes to the dispersity of PVA in the PLA matrix. The casting film is obtained by solvent evaporation, which hardly introduces any mechanical or temperature properties. Therefore, the miscibility change between PVA and PLA chains can be neglected for the casting film. With a better chain mobility, the crystallization of PLA is well defined in solution. The crystallinity of the PLA matrix declines with the addition of PVA, since the interaction between them hinders the movement of molecular chains. Meanwhile, PVA is still in a dispersed state without an external driving force, and it cannot crystallize (Figure S1b). Comparatively speaking, fast stretching during electrospinning promotes the aggregation of PVA chains, while hydrogen bonding is overcome (Figure 1a). Stretching also induces the crystallization of PVA chains partially, which is confirmed by the exothermic peaks at 200 °C in Figure 2c. The rapid evaporation of solvent during electrospinning helps to preserve the structure. For both PVA and PLA, the speed of electrospinning is so fast that the molecular chains cannot crystallize completely. The PLA and PVA structural network is formed by microcrystal and amorphous structures, while the latter can also be treated as a tie chain. Although a hydrogen bond is weaker than ionic and covalent bonds, it still links two kinds of molecular chains in the blended fiber. Therefore, a structure with two penetrated networks is formed during spinning. For the composite fiber, both of the structural networks contribute to the strength and toughness in observed in the tensile experiment.

## 4. Conclusions

To compare the effect of PVA on the tensile properties of PLA fiber, electrospun single fibers and the membrane of PLA/PVA with different PVA content (0%, 5%, 10%, and 20%) were prepared in this work. The decreased miscibility between PVA and PLA was first checked by DSC, and it is confirmed to decrease during electrospinning. However, both the toughness and the strength of the PLA membrane are found to be improved by the blending of PVA, whose content is less than 5% (*w*/*w*). PLA/PVA fibers with several sets of diameters were extracted to study the micro-mechanical properties. Regardless of the diameter, the fracture strain and Young’s modulus of the PLA/PVA fiber with a PVA content less than 10% (*w*/*w*) is higher than that of pure PLA fiber. The modulus of a single fiber, which is much larger than that of the membrane, exhibits a nonlinear monotonous decrease with the increase in fiber diameter, attributed to the voids in the membrane’s transverse section and the supramolecular structure. A model of the double networks, one microcrystal and one amorphous, is proposed to contribute to the improvement in strength and toughness. The successful development of a ductile and high-modulus PLA nanofiber scaffold will open a new era toward the designing of high-performance advanced biomaterials in the field of tissue engineering.

## Figures and Tables

**Table 1:** Detailed information obtained from DSC thermal analysis of PLA fiber and its composites.

Sample	*T*_cc_ (°C)	*T*_m_ (°C)	δ*H^PLA,cc^* (J/g)	δ*H^PLA,m^* (J/g)	*X_c_* (%)
**PLA**	85.9	168.0	10. 5	42.1	33.8
**L95V5**	89.5	166.8	10.9	32.4	26.5
**L90V10**	92.0	166.3	16.3	35.0	22.2
**L80V20**	94.7	163.4	15.9	28.8	17.3
**PVA**	/	224.6	/	0	/

**Table 2:** Parameters of the tensile properties of PLA and its composites.

Sample	Fracture Strain	Young’s Modulus (MPa)	Yield Stress (MPa)
**PLA**	0.8	50	1.6
**L95V5**	1.1	125	3.0
**L90V10**	0.20	129	3.1
**L80V20**	0.1	135	3.4

## Supplementary Materials

The following are available online at http://www.mdpi.com/2073-4360/10/10/1178/s1.
