# Supplementary material for: Simultaneous Enhancement of Strength and Toughness of PLA Induced by Miscibility Variation with PVA
Source: Polymers (Basel). 2018 Oct 22;10(10):1178. doi: 10.3390/polym10101178 (PMC6403973; doi:10.3390/polym10101178)
Supplement: Supplementary file 1 [file polymers-10-01178-s001.pdf]

Supporting Information

Simultaneous Enhancement of Strength and Toughness of PLA Induced by Miscibility Variation with PVA

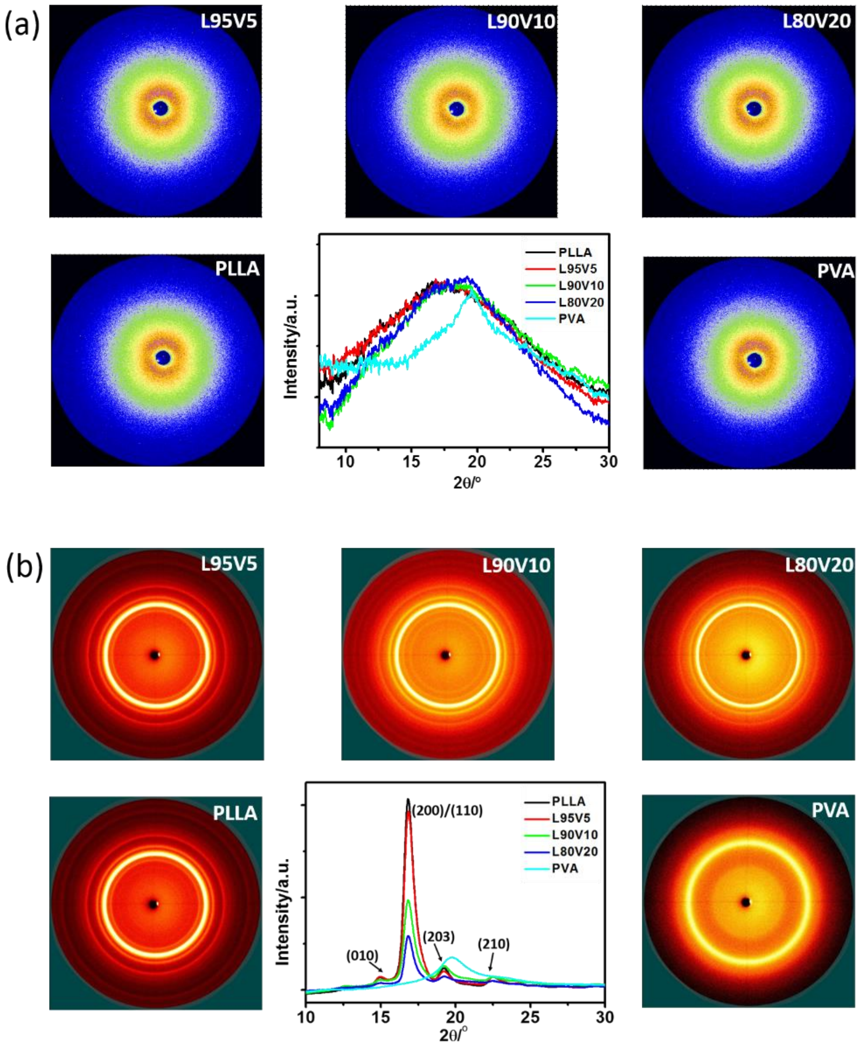

Figure S1. (**a**) The 2D WAXD images and the corresponding 1D curves of the PLA fiber membrane. (**b**) The WAXD images and curves of the PLA casting film.

The mechanical properties result of single fibers was fitted with the formula:

$$y = A_2 + (A_1\text{-}A_2)/[1 + (x/x_0)\text{^}p] \qquad \text{(S1)}$$

For Young's modulus of pure PLA fibers, the parameters in the formula are shown in Table S1, and the other fitting result also follows the formula with different parameters.

Table S1. Fitting parameters of Young's modulus of pure PLA

	A_1	A_2	x_0	p

Value	2044.16228	1.17317	0.01503	1.91332
Standard error	1.48022E6	0.72729	5.7634	2.49802

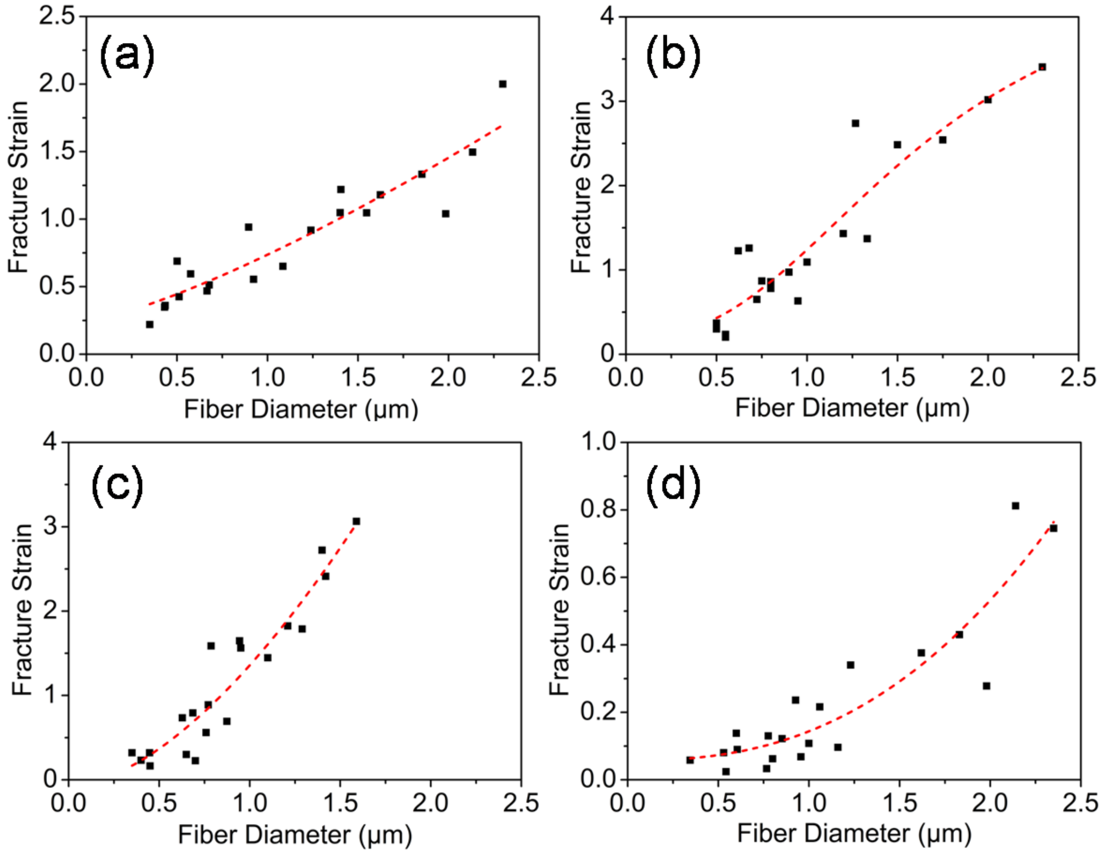

Figure S2. Fracture strain of PLA nanofibers with different PVA content: (**a**) PLA, (**b**) L95V5, (**c**) L90V10, (**d**) L80V20. The diameter of fibers varies from 0.3 μm to 2.5 μm and the dashed line represents the fitting result of 20 data points using the power function.

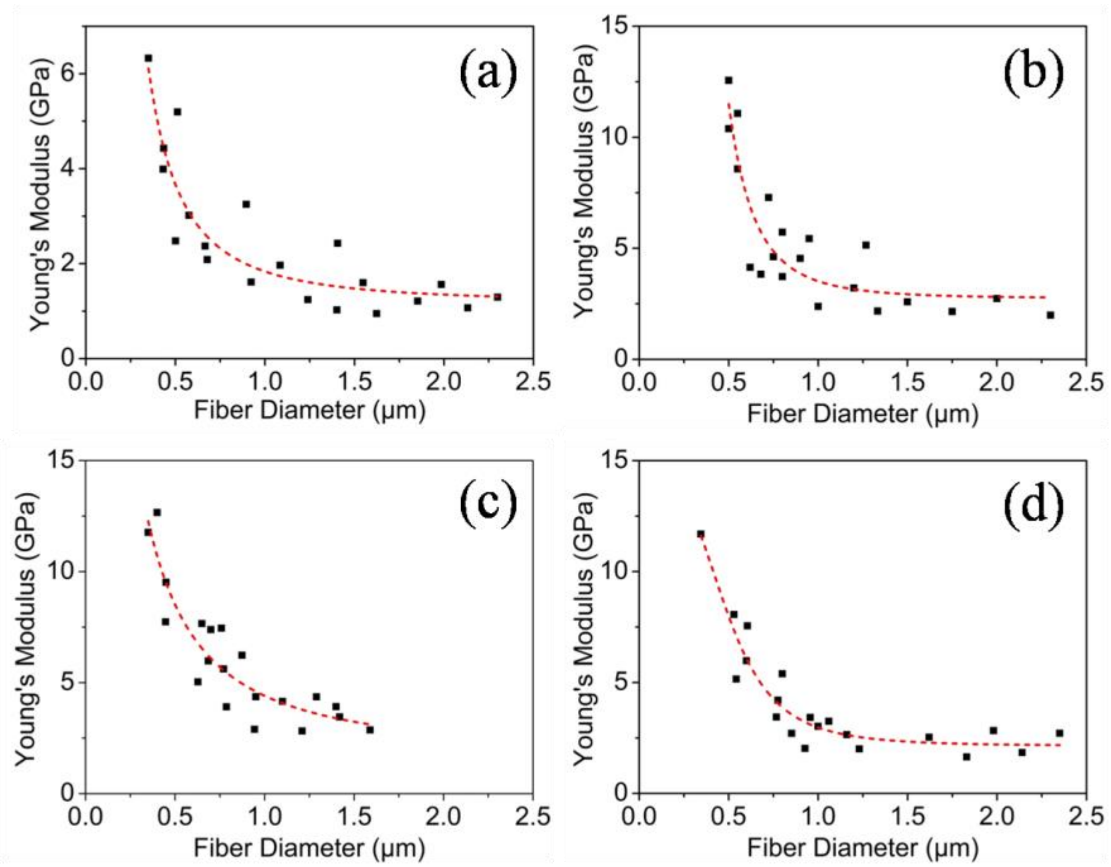

Figure S3. Young’s modulus of PLA nanofibers with different PVA content: (**a**) PLA (**b**) L95V5, (**c**) L90V10, (**d**) L80V20. The diameter of fibers varies from 0.3 μm to 2.5 μm and the dashed line represents the fitting result of 20 data points using the power function.
